# Structure of the Wechsler Intelligence Scale for Children – Fourth Edition in a Group of Children with ADHD

**DOI:** 10.3389/fpsyg.2016.00737

**Published:** 2016-05-30

**Authors:** Rapson Gomez, Alasdair Vance, Shaun D. Watson

**Affiliations:** ^1^School of Health Sciences and Psychology, Federation University Australia, BallaratVIC, Australia; ^2^Academic Child Psychiatry Unit, Department of Paediatrics, University of Melbourne, MelbourneVIC, Australia

**Keywords:** WISC-IV, ADHD, factor structure, bifactor model, academic performance

## Abstract

**Objective:** This study used confirmatory factor analysis to examine the factor structure for the 10 core WISC–IV subtests in a group of children (*N* = 812) with ADHD.

**Method**: The study examined oblique four- and five-factor models, higher order models with one general secondary factor and four and five primary factors, and a bifactor model with a general factor and four specific factors.

**Results**: The findings supported all models tested, with the bifactor model being the optimum model. For this model, only the general factor had high explained common variance and omega hierarchical value, and it predicted reading and arithmetic abilities.

**Conclusion:** The findings favor the use of the FSIQ scores of the WISC-IV, but not the subscale index scores.

## Introduction

Attention deficit/hyperactivity disorder (ADHD) is one of the most common childhood psychological disorders (diagnostic and statistical manual of mental disorders; DSM-5; American Psychiatric Association [APA], 2013). Major practice guidelines for ADHD, such as that of the American Academy of Child, and Adolescent Psychiatry [AACAP] (2007), have recommended intelligence testing for clinical evaluation of children with ADHD. The major reason for this is that as much as 70% of children with ADHD have comorbid learning disorders (Mayes et al., 2000; Mayes and Calhoun, 2006), and knowledge on an individual’s intellectual level can facilitate a better understanding of learning disorders. Additionally, as the Wechsler Intelligence Scale for Children-Fourth Edition (WISC-IV; Wechsler, 2003) is the most often used test of intelligence (Gresham and Witt, 1997), a comprehensive understanding of its factor structure in children with ADHD would be valuable as it could lead to a better understanding and more valid information on the intellectual, cognitive, and learning abilities of this group. The current study examined several structural models proposed for WISC-IV in a group of children and adolescents (henceforth referred to as children) with ADHD.

The WISC-IV measures intellectual ability of children from 6 to 16 years. It was developed to provide an overall measure of general cognitive ability, and also measures of intellectual functioning in Verbal Comprehension (VC), Perceptual Reasoning (PR), Working Memory (WM) and Processing Speed (PS). The VC, PR, WM, and PS subscales provide scores for the Verbal Comprehension Index (VCI), the Perceptual Reasoning Index (PRI), the Working Memory Index (WMI), and the Processing Speed Index (PSI), respectively. Together, the VCI, PRI, WMI, and PSI provide the overall level of intelligence, or Full Scale IQ (FSIQ). Although the full version of the WISC-IV has 15 subtests, only ten are considered core, and used more often when testing intelligence (Wechsler, 2003). The core subtests for VC are Vocabulary, Similarities, and Comprehension. The core subtests for PR are Block Design, Picture Concepts, and Matrix Reasoning. The core subtests for WM are Digit Span and Letter-Number Sequencing, and the core subtests for PR are Coding and Symbol Search. The remaining five subtests, which are referred to as supplementary subtests, are Information and Word Reasoning (part of VC), Picture Completion (part of PR), Arithmetic (part of WM), and Cancelation (part of PS).

**FIGURE 1:**
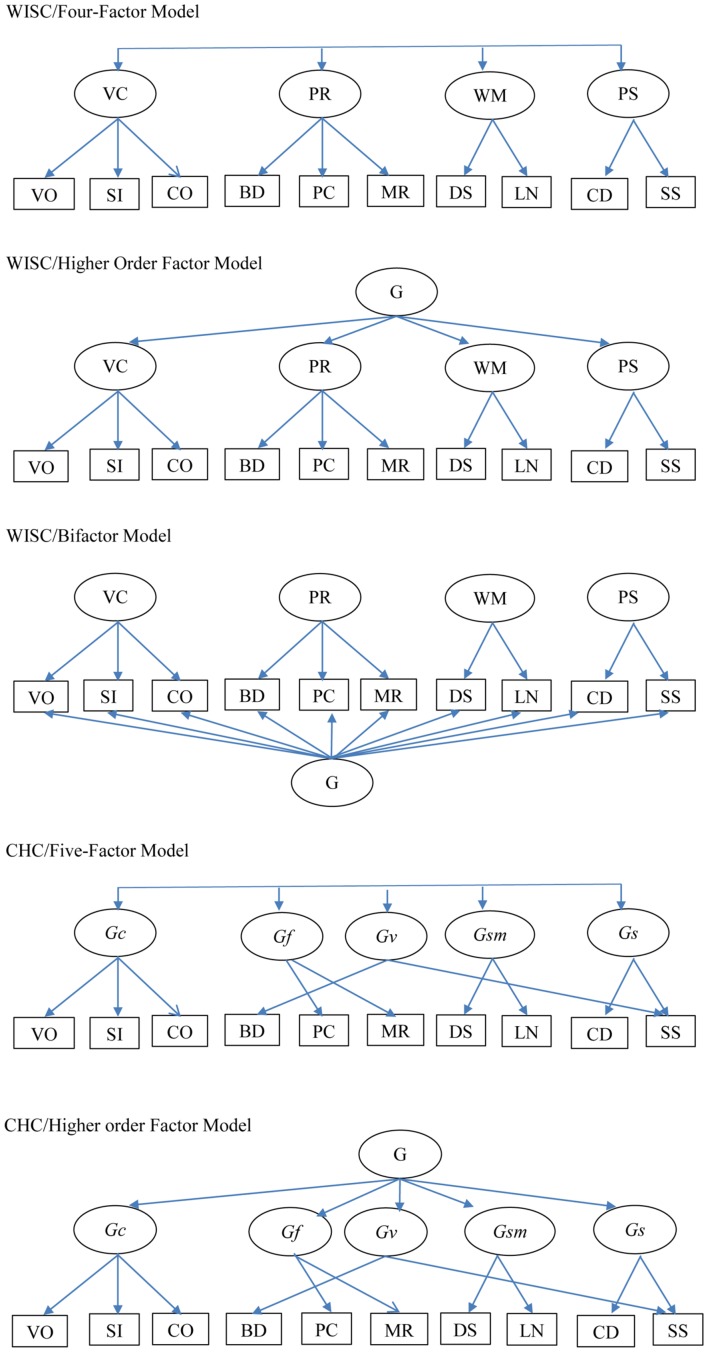
**Factor models examined in the study.** G, general factor; VC, Verbal Comprehension; PR, Perceptual Reasoning; WM, Working Memory; PS, Processing Speed; *Gc*, crystallized intelligence; *Gf*, fluid reasoning; *Gv*, visual processing; *Gsm*, short-term memory; *Gs*, processing speed; VO, Vocabulary; SI, Similarities; CO, Comprehension; BD, Block Design; PC, Picture Concept; MR, Matrix Reasoning; DS, Digit Span; LN, Letter-Number Sequencing; CD, Coding; SS, Symbol Search.

The factor structure for the core subtests of the WISC-IV has been examined in a number of studies involving general community and clinic-referred children, including those with learning disorders (e.g., Wechsler, 2003; Keith, 2005; Watkins et al., 2006; Sattler, 2008; Bodin et al., 2009; Watkins, 2010; Devena et al., 2013; Nakano and Watkins, 2013; Watkins et al., 2013; Canivez, 2014; Styck and Watkins, 2016). Across these studies, support has been reported for an oblique four-factor model, a higher order factor model, and a bifactor model. The oblique four-factor model has factors for VC, PR, WM, and PS, corresponding to the subscales for VC, PR, WM, and PS. The higher order factor model has first-order factors for VC, PR, WM, and PS, and a single higher order general factor. In this model, the general factor captures the common variances of all first-order factors, and the first-order factors capture the covariance across the subtests comprising the factors. The bifactor model is an orthogonal model, with five primary factors. In this model, all subtests load on a general factor, and each subtest loads on its own specific factor (VC, PR, WM, or PS). The general factor captures the covariance of all subtests, and the VC, PR, WM, and PS specific factors capture the unique covariance of the subtests within them after removing the covariance captured by the general factor. Thus the specific factors capture their unique variance. The oblique four-factor, the higher order factor, and the bifactor models are shown in **Figure 1.** With the exception of the study by Nakano and Watkins (2013), the other studies that have compared the oblique four-factor model, the higher order factor model, and the bifactor model have reported more support for the bifactor model than the four-factor oblique model and the higher order factor model (Watkins, 2010; Devena et al., 2013; Watkins et al., 2013; Canivez, 2014; Styck and Watkins, 2016). Nakano and Watkins reported most support for the higher order factor model, although it differed minimally from the bifactor model.

For the WISC-IV bifactor model, a number of past studies have reported on the explained common variance (ECV; Reise et al., 2013a), as well as the omega hierarchical (ω_h_) and omega subtests (ω_s_; McDonald, 1999; Zinbarg et al., 2005) of the general and specific factors, respectively. The ECV of a general factor is the common variance explained by the general factor divided by the total common variance. The ECV of a specific factor is the common variance explained by the specific factor divided by the total common variance. The ECV of the general factor will be high whenever there is little common variance beyond that of the general factor. Thus high values indicate the presence of a general dimension in the bifactor model (Reise et al., 2013a). The ω_h_ value of the general factor can be interpreted as a model-based index of the internal consistency reliability of the total scale (Brunner et al., 2012). The ω_h_ can also be interpreted as an estimator of how much variance in summed (standardized) scores can be attributed to the single general factor (McDonald, 1999). It is obtained by dividing the amount of variance explained by the general factor in a scale by the total amount of variance explained by all the items in the scale. The ω_s_ value of a specific factor can be interpreted as a model-based index of the internal consistency reliability of the specific scale, and an estimator of how much variance in summed (standardized) scores can be attributed to the specific factor (McDonald, 1999; Brunner et al., 2012). It is computed by dividing the amount of variance explained by the specific factor by the total amount of variance explained by all the items in the scale. The values for ω_h_ and ω_s_ range from 0 to 1, with 0 indicating no reliability and 1 reflecting perfect reliability. According to Reise et al. (2013a), ω_h_ and ω_s_ values of at least 0.75 are preferred for meaningful interpretation of a scale.

Existing data for the WISC-IV bifactor model show that the ECV of the general factor is between 2 and 3 times more than the combined ECV of the specific factors, with virtually all variance in the subtests being explained much more by the general factor than the respective specific factors (Watkins, 2010; Devena et al., 2013; Watkins et al., 2013; Canivez, 2014; Styck and Watkins, 2016). Also, the ω_h_ of the general factor is much higher (ranging from 0.67 to 0.87) than the ω_s_ values of the four specific factors (ranging from 0.10 to 0.53; Watkins, 2010; Devena et al., 2013; Watkins et al., 2013; Canivez, 2014; Styck and Watkins, 2016), adding support for the utilization of the FSIQ score over the index scores. These findings indicate support for the presence of a general dimension in the bifactor model, and that only the general factor can be meaningfully interpreted. They support the utilization of the total score, and not index scores of the WISC-IV.

For the core subtests, at least three studies have examined the factor structure of the WISC-IV for groups of children with ADHD (Yang et al., 2013; Styck and Watkins, 2014; Thaler et al., 2015). Across all these studies, support was found for the four-factor oblique model. Support was also found for the higher order factor model (Styck and Watkins, 2014). Although the study by Styck and Watkins (2014) reported good fit for the bifactor model, this model was rejected as it had an inadmissible solution (one residual variance was negative). The study by Thaler et al. (2015) found support for two five-factor oblique models, based on the Cattell–Horn–Carroll (CHC) theoretical model of intelligence (McGrew, 2005). One of these models, called here the oblique five-factor SS model, comprises factors for crystallized intelligence (*Gc*; comprising Vocabulary, Similarities, and Comprehension), fluid reasoning (*Gf*; comprising Picture Concepts and Matrix Reasoning), visual processing (*Gv*; comprising Block Design and Symbol Search), short-term memory (*Gsm*; comprising Digit Span and Letter–Number Sequencing), and PS (*Gs*; comprising Symbol Search and Coding). This model is also shown in **Figure 1.** The second model, called here the oblique five-factor MR-SS model, differed from the other oblique five-factor SS model by specifying the Matrix Reasoning subtest to cross-load onto the *Gv* factor. As will be evident, both of the CHC models are not similar to the WISC-IV inspired oblique four-factor, higher order or bifactor models.

In terms of comparison of models, Styck and Watkins (2014) found better fit for the higher order factor model than the oblique four-factor model. Thaler et al. (2015) found that both their five-factor models had better fit than the oblique four-factor model. Although the oblique five-factor MR-SS model showed marginally better fit than the oblique five-factor SS model, the oblique five-factor SS model was adopted as the better model as the Matrix Reasoning subtest in the oblique five-factor MR-SS model did not load significantly on the *Gv* factor. For the higher order factor model tested by Styck and Watkins (2014), and for the higher order oblique five-factor SS model reported by Thaler et al. (2015) the general factor explained more variance than the specific factors for all subtests, with the exception of Coding and Symbol Search (both PS subtests). In the study by Styck and Watkins (2014), both Coding and Symbol Search had about equal on the general and PS specific factors. In the study by Thaler et al. (2015) Symbol Search loaded equally on the general factor and its own (PS) specific factor, and Coding had a higher loading on its own specific factor (PS). For the higher factor models in the and Styck and Watkins (2014) and Thaler et al. (2015) studies, the ECV of the general factor was about twice the total ECV of all the specific factors together. Styck and Watkins (2014) also reported that the ω_h_ value for the general factor (0.78) was much higher than the ω_s_ values of the four specific factors (ranging from 0.09 to 0.34), thereby indicating that only the FSIQ had sound reliability.

Overall, therefore, most of the findings for the factor structure of the core WISC-IV subtests in children with ADHD are comparable with existing data involving the general community and clinic-referred children, including those with learning disorders (Watkins et al., 2006, 2013; Watkins, 2010; Devena et al., 2013; Nakano and Watkins, 2013; Canivez, 2014; Styck and Watkins, 2016). Across these studies, support has been reported for the oblique four-factor model and the higher order factor model. The CHC based five-factor oblique model and a higher order structure of this model have also been supported when all 15 (core and supplementary) WISC-IV subtests were examined (Keith et al., 2006; Chen et al., 2009; Golay et al., 2013).

Despite the similarities in the findings on children with ADHD and children from the general community and clinics, we wish to argue that there are limitations in existing findings on the factor structure of the WISC-IV in children with ADHD. First, there has only been three studies involving children with ADHD (Yang et al., 2013; Styck and Watkins, 2014; Thaler et al., 2015), with only one study reporting on the applicability of the higher order factor model (Styck and Watkins, 2014), and the oblique five-factor model (Thaler et al., 2015). Second, the study by Styck and Watkins (2014), the only study that has tested the applicability of the bifactor model for children with ADHD, did not find an admissible solution for this model. As this study used a small sample (*N* = 233), it is possible that with 30 parameters to be estimated in the bifactor model, this could have contributed to the inadmissible solution. Given the generally robust support for the bifactor model in community and clinic-referred samples, it is conceivable that with larger sample sizes, the bifactor model will also be supported for children with ADHD. Third, as pointed out by Styck and Watkins (2014), the relevance of all their findings for children with ADHD is uncertain. This is because as school multidisciplinary evaluation teams were responsible for making eligibility decisions that had to adhere to the Individuals With Disabilities Education Improvement Act (2004), the children identified as having ADHD may not be comparable to samples of children with ADHD diagnosed in conventional child mental health clinics. Fourth, as the study by Styck and Watkins (2014) did not consider the medication status of participants, it cannot be ruled out that their findings were not confounded by medication effects, as long-term use of medication has been shown to influence the IQ of children with ADHD (Gillberg et al., 1997; Gimpel et al., 2005).

Another limitation is that although IQ has consistently been shown to be associated with academic achievement (Naglieri and Bornstein, 2003), at present, no study has examined the predictive validity of the factors in the bifactor model of the WISC-IV, modeled in terms of a general factor and the specific factors representing the index scales (VC, PR, WM, and PS). Using multiple regression analysis of observed scores for WISC-IV FSIQ, VCI, PRI, WMI, and PSI, studies involving non-ADHD samples have reported that the index scores provide only slightly additional variance in the prediction of academic achievement scores, including reading and arithmetic (Glutting et al., 2006; Canivez et al., 2014). A recent study (Beaujean et al., 2014), also with a non-ADHD sample that modeled all core and supplementary tests of WISC-IV in terms of the Cattell–Horn–Carroll (CHC; Schneider and McGrew, 2012) theory of cognitive abilities showed that the general factor had a stronger association with reading and arithmetic than any of the specific factors. Similar findings have been reported for the Wechsler Adult Intelligence Scale- Fourth Edition (WAIS-IV; Wechsler, 2008) bifactor model with a general factor and the factors for the index scales (Kranzler et al., 2015). Based on these findings, it can be speculated that the general intelligence factor would also be associated with the academic abilities of children with ADHD. However, as low reading ability (Gathercole et al., 2006; Alloway et al., 2009; Alloway and Alloway, 2010) and arithmetic ability (Bull and Scerif, 2001; Swanson and Sachse-Lee, 2001) have been linked to poor WM, and as WM defects have been strongly associated with ADHD (Martinussen et al., 2005; Willcutt et al., 2005; Walshaw et al., 2010), the WM specific factor (which captures WM ability that is independent of the general intelligence) may also be associated with academic achievement abilities.

Given existing limitations, the first aim of the current study was to examine the factor structure of the ten WISC–IV core subtests in a large group (*N* = 812) of children with ADHD, all directly diagnosed using DSM-IV TR (American Psychiatric Association [APA], 2000) ADHD criteria. As they were new to mental health services, none of them had not been on medication, and were not on medication at any time before or during testing. Consistent with models previously supported, the study examined the following models: an oblique first-order four-factor model, the CHC-based oblique five-factor SS model, higher order and bifactor models based on the four-factor model, and a higher order factor model based on the five-factor SS model. Since cross-factor pattern coefficients are not allowed in a CFA bifactor model (as it distorts parameter estimates; Rios and Wells, 2014), the equivalent bifactor version of the five-factor SS model was not tested as it has cross-loadings for Symbol Search. To reduce confusion, the oblique four-factor and five-factor models will be referred to as WISC/four-factor model and CHC/five-factor model, respectively; the higher order factor models with four and five primary factors will be referred to as WISC/higher order factor model and CHC/higher order factor model, respectively; and the bifactor models with four specific factors will be referred to as WISC/bifactor model. The five models tested are depicted in **Figure 1.** The second aim of the study was to examine the ECV, and model-based internal consistency reliability for factors in the model selected as the optimum model. The third aim was to examine how the general and specific factors in the bifactor model predicted reading and arithmetic. Based on previous findings involving children with ADHD and children in general, we predicted support for all the models tested, with the WISC/bifactor model being the best fitting model. For this model, we expected that with the exception of Coding and Symbol Search, the factor pattern coefficients of the subtests on the general factors would be relatively higher than on the specific factors. We also expected that the ECV and ω_h_ values for the general factor would be relatively higher than the ECV and ω_s_ values for the specific factors. We also expected the general factor and the WM specific factors to predict reading and arithmetic abilities.

## Materials and Methods

### Participants

The data for all participants were collected archivally from the (omitted for blind review). The (omitted for blind review) is an out-patient psychiatric unit that provides services for children and adolescents with behavioral, emotional, and learning problems. The study was approved by the (omitted for blind review) ethics committee as part of our group’s comprehensive examination of executive function in children and adolescents with ADHD including comorbid disorders. Each legal guardian and participant provided informed written consent for any data provided by them to be used in future ethics approved research studies. This is a standard part of the (omitted for blind review) assessment procedure. For the current study we used the records of children, aged between 6 and 16 years, referred between 2004 and 2012. In total, there were 812 children with ADHD, comprising 522 (64.3%) combined type, 227 (28.0%) inattentive type, and 63 (7.8%) hyperactivity/impulsive type. There were 75.9% males (*N* = 616). The overall mean age of participants was 11.03 years (*SD* = 3.08). All children were naive with respect to stimulant and other psychoactive medications at the time of testing.

All children were diagnosed using the Anxiety Disorders Interview Schedule for Children (ADISC-IV; Silverman and Albano, 1996), a semi-structured interview schedule with sound psychometric properties (Silverman et al., 2001). The frequencies (percentages) of any anxiety disorder (Separation Anxiety, Social Phobia, Specific Phobia, Panic, Agoraphobia, Generalized Anxiety, Obsessive Compulsive and/or Post-Traumatic Stress Disorders), any depressive disorder (Dysthymic and/or Major Depressive Disorders), and Oppositional Defiant Disorder (ODD) were 604 (74.4%), 348 (42.9%), and 612 (75.4%), respectively. In all, 59 (7.4%) were not comorbid for any other disorder, and 217 (26.7%), 261 (32.1%), and 275 (33.9%) had one, two, and three additional disorders, respectively.

The percentages of father employment status were as follows: employed = 79.0%, home duties = 2.5%, pensioner/retired = 4.7%, unemployed = 9.1%, others/unknown = 4.7%. The percentages of highest father education level were as follows: tertiary = 16.0%, high school/some years in secondary school or equivalent = 62.8%, technical certificate or equivalent = 19.0%, primary school = 2.0%, and no schooling = 0.2%. Thus, most fathers of participants were employed, and more than two-third of participants had fathers who had attended at least secondary school. In terms of parental relationship, 54.1% of parents were living together.

### Materials and Procedure

The measures included in this study were the Anxiety Disorders Interview Schedule for Children (ADISC-IV; Silverman and Albano, 1996), the Wechsler Intelligence Scale for Children – Fourth Edition (WISC-IV; Wechsler, 2003), and the Wide Range Achievement Test-3 (WRAT-3; Wilkinson, 1993).

#### Anxiety Disorders Interview Schedule for Children (ADISC-IV; Silverman and Albano, 1996)

The ADISC-IV is a semi-structured interview, based on the DSM-IV diagnostic system. Although ADISC-IV has been designed primarily to facilitate the diagnosis of the major anxiety and depressive disorders, it can also be used for diagnosing other major childhood disorders, including ADHD. Although the diagnosis of ADHD requires the presence of cross-situational symptoms, in this study diagnosis was based on parent interviews alone. There is support for the concurrent validity of the ADISC-IV ADHD module based on parent interviews (Jarrett et al., 2007). The ADISC-IV guidelines for diagnosis are that the child be given a diagnosis of all disorders meeting the diagnostic criteria. Disorders are diagnosed categorically (either present or absent). The scores of ADISC-IV have sound psychometric properties, including excellent test-retest reliability over a 7–14 days interval (Silverman et al., 2001). Kappa values for interview with parents ranged from 0.65 to 1.00 (Silverman et al., 2001). For the current study, there was adequate inter-rater reliability for the diagnoses made between the research assistants (who collected the data) and their supervisors (who supervised the data collection), and also between research assistants who administered the ADISC-IV (kappa values generally more than 0.88, and ranged from 0.82 to 0.95).

#### Wechsler Intelligence Scale for Children – Fourth Edition (Wechsler, 2003)

The WISC-IV is a test of intellectual ability for children ages 6 to 16 years. It is individually administered, and has 15 subtests. Each subtest is allocated to either the VC, PR, WM, or PS subscales. Each subscale has a standardized mean and *SD* of 100 and 15, respectively. The FSIQ is composed of 10 core subtests: three VC (Vocabulary, Similarities, and Comprehension), three PR (Block Design, Picture Concepts, and Matrix Reasoning), two WM (Digit Span and Letter-Number Sequencing), and two PS (Coding and Symbol Search), and has a standardized mean and *SD* of 100 and 15, respectively. The FSIQ and the four indices, as well as the subtests, have excellent reliability (i.e., internal consistency and test–retest) and validity (Wechsler, 2003; Williams et al., 2003). The original WISC-IV was standardized using a nationally representative sample of the U.S. population, with subsequent publication of Australian norms (Wechsler, 2005). The WISC-IV scores in this paper are based on the latter.

#### Wide Range Achievement Test-3 (WRAT-3; Wilkinson, 1993)

Reading and arithmetic abilities were measured using the WRAT-3. The reading test in the WRAT-3 has letters and individual words that the individual has to name or pronounce. The arithmetic test has two parts. The first part covers counting, reading number symbols, and verbally presented simple arithmetic problems. The second requires the individual to calculate up to 40 arithmetic problems. This is a paper and pencil task. Both the reading and arithmetic tests have sound reliability and validity (Wilkinson, 1993).

### Procedure

Each participant and their parents were interviewed separately in testing sessions over two consecutive days. Breaks were provided as needed. Parental consent forms were completed prior to the assessment. The data collected covered a comprehensive demographic, medical (primarily neurological and endocrinological), educational, psychological, familial, and social assessment of the child and their family based on information obtained from parents and children. Standard procedures were used for the administration of all measures, including the ADISC-IV and WISC-IV. Where necessary, researchers read the items to participants who then completed their responses. Approximately 95% of the parent ADISC-IV interviews involved mothers only, and the rest involved fathers only or both fathers and mothers together. Clinical diagnosis, based on the ADISC-IV, was determined by two consultant child and adolescent psychiatrists who independently reviewed these data. The inter-rater reliability for diagnoses of the two psychiatrists was high (kappa = 0.90).

All psychological tasks were administered by research assistants, who were advanced masters or doctoral students in clinical psychology, and under the supervision of registered clinical psychologists. The research assistants were provided with extensive supervised training and practice by the psychologists prior to them collecting data. This training for the ADISC-IV included observations of it being administered by the registered psychologists. The research assistants commenced administering the ADISC-IV once they had attained competence in its administration, as assessed by the registered psychologists.

### Analytical Procedure

All CFA models in the study were conducted using maximum likelihood (ML) estimation in M*plus* (Version 7; Muthén and Muthén, 2012). The ML procedure indicates statistical fit in terms of ML χ^2^ values. However, as χ^2^ values are inflated by large sample sizes, the fit of the models was examined using three approximate fit indices: root mean squared error of approximation (RMSEA), comparative fit index (CFI), and Tucker-Lewis Index (TLI). The guidelines suggested by Hu and Bentler (1998) are that RMSEA values close to 0.06 or below be taken as good fit, 0.07 to <0.08 as moderate fit, 0.08 to 0.10 as marginal fit, and >0.10 as poor fit. For the CFI and TLI, values of 0.95 or above are taken as indicating good model-data fit, and values of 0.90 and <0.95 are taken as acceptable fit. As not all the models in the study are nested (Canivez, 2014; Watkins et al., 2013), meaningful differences between well-fitting models were examined in terms of ΔCFI of 0.002 or higher (Meade et al., 2008). The Akaike information criterion (AIC) values were also used. The AIC considers statistical goodness-of-fit as well as model parsimony, with smaller values representing a better fit.

In order to examine the predictions of reading and arithmetic by the factors in the bifactor model, this model was extended to include variables for WRAT-3 reading and arithmetic, and reading and arithmetic were regressed on the WISC-IV factors.

## Results

### Missing Values, and Mean (SD) of the WISC-IV Subtests and Scales

There were no missing values in the data set. The standardized mean (*SD*) scores for 10 WISC-IV subtests are shown in **Table 1.** The standardized mean (*SD*) for FSIQ, VCI, PRI, WMI, and PSI were 87.56 (15.08), 89.25 (15.31), 93.00 (15.29), 87.70 (15.24), and 87.40 (14.95), respectively.

**Table 1:** Mean, standard deviation, skewness, and kurtosis of the WISC-IV core subtests.

Subtests	Mean	Standard deviation	Skewness	Kurtosis
Vocabulary	7.87	2.87	0.23^∗∗^	0.60^∗∗^
Similarities	8.39	3.18	0.07	-0.21
Comprehension	8.19	3.13	-0.06	0.18
Block design	8.83	3.03	0.05	-0.20
Picture concepts	8.96	3.14	-0.24^∗^	0.19
Matrix reasoning	8.71	3.00	0.09	-0.10
Digit span	8.49	3.15	0.08	0.26
L–N sequencing	8.05	3.12	-0.35^∗∗∗^	-0.21
Coding	7.33	3.23	0.46^∗∗∗^	0.41^∗^
Symbol search	8.11	2.93	-0.23^∗∗^	0.15


*^∗^*p* < 0.05, ^∗∗^*p* < 0.01, ^∗∗∗^*p* < 0.001.*

### Confirmatory Factor Analysis of All WISC-IV Models Tested

**Table 2** shows the results of all the CFA models tested. Based on guidelines for goodness-of-fit values (RMSEA, CFI, and TLI) proposed by Hu and Bentler (1998), there was good fit for the WISC/four-factor and CHC/five factor models. The AIC value of the CHC/five factor model was lower than all other models, implying that this could be the best fitting model. For this model, however, the correlations of the factors were all statistically significant, ranging from 0.42 (*Gs* with *Gc*) to 0.81 (*Gv* with *Gf*), with 8 of the 10 correlations being >0.50. For the WISC/four-factor model, the correlations of the factors were all statistically significant, ranging from 0.42 (PS with VC) to 0.77 (PR with VC). These high correlations are strongly indicative of a higher order or a hierarchical factor model, including a bifactor model (Gorsuch, 1983; Thompson, 2004; Canivez, in press). While the CFI and TLI values for the WISC/higher order factor model and the CHC/higher order factor model showed good fit, the RMSEA values showed only moderate fit. These models had highly comparable AIC values (38212 for the WISC/higher order factor model, and 38213 for the CHC/higher order factor model). For the WISC/bifactor model, all the goodness-of-fit values (RMSEA, CFI, and TLI) indicated good fit, and this model showed better fit than the WISC/higher order factor model (ΔCFI = 0.003), and CHC/higher order factor model (ΔAIC = -14). Also the AIC value for the WISC/bifactor model was lower than both the WISC/higher order factor model (ΔAIC = -13), and CHC/higher order factor model (ΔAIC = -14). Thus, although several models showed acceptable fit, the WISC/bifactor model was taken as the optimum model for the 10 core WISC-IV subtests as it showed the best fit. Given this, we examined the factor pattern coefficients, ECV, ω_h_, and ω_s_ values for the factors in this model, and how the factors in this model predicted reading and arithmetic abilities.

**Table 2:** Fit of all the factor models tested in the study.

Model	χ^2^	df	RMSEA (90% CI)	CFI	TLI	AIC
WISC/Four-factor	107.42^∗∗∗^	29	0.058 (0.046–0.070)	0.976	0.96	38185
WISC/Higher order factor	137.84^∗∗∗^	31	0.065 (0.054–0.076)	0.967	0.95	38212
WISC/Bifactor	116.95^∗∗∗^	27	0.064 (0.052–0.076)	0.972	0.94	38199
CHC/Five-factor	79.85^∗∗∗^	24	0.054 (0.041–0.067)	0.983	0.97	38168
CHC/Higher order factor	135.01^∗∗∗^	29	0.067 (0.056–0.079)	0.968	0.95	38213


*All χ^2^ goodness-of-fit tests were statistically significant at *p* < 0.001. CFI, comparative fit index; CI, confidence interval; RMSEA, root mean square error of approximation; TLI, Tucker Lewis Index; AIC, Akaike information criterion. For the WISC/bifactor model, the factor pattern coefficients of the subtests within WM and PS (Coding and Symbol Search) were constrained to be equal. Without these modifications the bifactor model showed non-identification. ^∗∗∗^*p* < 0.001.*

### Factor Pattern Coefficients for the WISC/Bifactor Model

**Table 3** presents the completely standardized factor pattern coefficients of the ten subtests on the general and specific factors in the WISC/bifactor model. As indicated, for the general factor, all subtests showed statistically significant and salient factor pattern coefficients (ranging from 0.43 to 0.73), using Thurstone’s (1947) classical criterion for “salience” as standardized loading ≥0.3. Statistical significant and salient factor pattern coefficients were also found for all subtests for VC (ranging from 0.42 to 0.51), WM (0.35 for both subtests), and PS (0.57 for both subtests). For all three PR subtests, the factor pattern coefficients were not statistically significant or salient (ranging from 0.05 to 0.13). In an absolute sense, except the two subtests for PS (Coding and Symbol Search), all the factor pattern coefficients were higher for the general than the specific factors.

**Table 3:** Factor pattern coefficients and sources of variance in the WISC/bifactor model.

	General		VC		PR		WM		PS			
							
	λ	Var	λ	Var	λ	Var	λ	Var	λ	Var	*h*^2^	*u*^2^
Vocabulary	0.73	0.53	0.51	0.26							0.79	0.22
Similarities	0.70	0.49	0.42	0.17							0.67	0.33
Comprehension	0.56	0.31	0.51	0.26							0.57	0.43
Block design	0.63	0.40			0.13	0.02					0.42	0.58
Picture concepts	0.64	0.40			0.05	0.00					0.41	0.59
Matrix reasoning	0.70	0.49			0.10	0.01					0.50	0.50
Digit span	0.57	0.32					0.35	0.12			0.45	0.55
L–N sequencing	0.67	0.45					0.35	0.12			0.57	0.43
Coding	0.43	0.19							0.57	0.33	0.51	0.49
Symbol search	0.48	0.23							0.57	0.33	0.56	0.44
Total factor pattern coefficients^2^	37.2		2.0		0.1		0.5		1.3			
Total uniqueness												4.6
ω*_h_*	0.81											
ω*_s_*			0.29		0.02		0.17		0.43			
ECV	0.70		0.13		0.00		0.05		0.12			
Cronbach’s alpha	0.87		0.85		0.74		0.67		0.69			


*λ, factor loading; Var, percentage of variance explained; VC, Verbal Comprehension; PR, Perceptual Reasoning; WM, Working Memory; PS, Processing Speed; L–N, Letter-Number; *h*^2^, communality; *u*^2^, uniqueness; ECV, Explained Common Variance; ω_h_, omega hierarchical; ω_s_, omega-subscale.*

### ECV, ω_h_, and ω_s_ Values for the factors in the WISC/Bifactor Model

**Table 3** also includes the ECV, ω_h_, and ω_s_ values for the WISC/bifactor model. As shown, the ECV for the general factor was 0.70. The ECV for the specific factors for VC, PR, WM, and PS were 0.13, 0.00, 0.05, and 0.12, respectively. The ω_h_ value for the full test (i.e., FSIQ) was 0.81. The ω_s_ values for VC, PR, WM, and PS subscales were 0.29, 0.02, 0.17, and 0.43, respectively. All these findings indicate support for a general factor and the utilization of the FSIQ score over the index scores.

### Predictions of Reading and Arithmetic by the Factors in the WISC/Bifactor Model

**Table 4** shows the path coefficients for the predictions of reading and arithmetic by the factors in the WISC/bifactor model. As shown, the general factor and WM specific factor predicted reading and arithmetic positively. The VC, PR, and PS specific factors did not predict reading or arithmetic.

**Table 4:** Standardized path coefficients for the predictions of reading and arithmetic by the factors in the WISC/bifactor model.

	Reading	Arithmetic
General	0.57^∗∗∗^	0.61^∗∗∗^
Verbal comprehension	0.20	0.12
Perceptual reasoning	0.01	0.14
Working memory	0.33^∗∗^	0.36^∗∗^
Processing speed	0.06	0.17


*^∗∗^*p* < 0.01, ^∗∗∗^*p* < 0.001.*

## Discussion

One aim of the study was to examine the applicability of the WISC/four-factor model, CHC/five-factor model, WISC/higher order model, CHC/ higher order model, and WISC/bifactor model for the 10 WISC–IV core subtests in a group of children with ADHD. As predicted, the findings supported all five models. The correlations among the factors in the WISC/four-factor and CHC/five-factor models were all high (ranging from 0.42 to 0.81), and the factor pattern coefficients of the primary factors on the general factor in the WISC/higher order factor and CHC/higher order factor models were also high (ranging from 0.64 to 0.99). Such high correlations suggest more preference for either the WISC/higher order factor model, CHC/higher order factor model, or WISC/bifactor model. Between these models, the WISC/bifactor model showed better fit. Thus despite the good fit for all models tested, the WISC/bifactor model can be considered more preferable than the WISC/ higher order factor model, or the CHC/higher order factor model.

Consistent with our findings, past studies involving children with ADHD have also reported support for the WISC/four-factor model (Yang et al., 2013; Styck and Watkins, 2014; Thaler et al., 2015), CHC/five-factor model (Thaler et al., 2015), and WISC/higher order model (Styck and Watkins, 2014). Also, previous studies involving the 10 core WISC subtests with the general community, clinic-referred samples, and children with learning disorders, have supported the WISC/four-factor, WISC/higher order factor, and WISC/bifactor models, with better support for the WISC/bifactor model (Watkins et al., 2006, 2013; Watkins, 2010; Devena et al., 2013; Nakano and Watkins, 2013; Canivez, 2014; Styck and Watkins, 2016). For all 15 subtests, support has also been reported for the CHC/five-factor model and CHC/ higher order factor model (Chen et al., 2009; Keith et al., 2006; Golay et al., 2013).

For the WISC/bifactor in the current study, all subtests showed statistically significant and salient factor pattern coefficients on the general factor. Although eight subtests also showed statistically significant and salient factor pattern coefficients on their specific factors, in an absolute sense, except for two subtests, all the factor pattern coefficients were higher on the general than the specific factors. The ECV values for the general factors were much higher (0.70) than that for the specific factors (ranging from 0.00 to 0.12). The ω_h_ value for the general factor in this model was also much higher (0.81) than the ω_s_ values of the specific factors (ranging from 0.02 to 0.43). Thus, the WISC/bifactor model can be considered an optimum model to represent the factor structure of the 10 core WISC-IV subtests. These findings were as expected and are consistent with existing data involving children in general (Devena et al., 2013; Watkins et al., 2013; Canivez, 2014).

Although our findings are highly comparable with existing data, they also extend existing data. This is the first study to demonstrate support for the WISC/bifactor model for the 10 WISC–IV core subtests in a group of children with ADHD. Although Styck and Watkins (2014) did not find an admissible solution for this model, our findings supportive of this model are likely to be more accurate. It is possible that the sample size (*N* = 233) in the Styck and Watkins (2014) study may have been too low for estimating this model (with 30 parameters to be estimated). Our findings are also likely to be more relevant for ADHD than the findings reported by Styck and Watkins (2014). While Styck and Watkins (2014) did not screen for medication used by participants, all participants in the current study were medication-free at the time of testing. Also, as noted by Styck and Watkins (2014), the ADHD diagnosis in their study may not have adhered to the standard diagnostic criteria, such as in DSM-5 (American Psychiatric Association [APA], 2013), as diagnosis had to also adhere to the Individuals With Disabilities Education Improvement Act (2004).

A third new finding in the current study is in relation to how the factors in the bifactor predict reading and arithmetic performance. The findings showed that the general factor and the WM specific factor predicted reading and arithmetic ability. None of the specific factors predicted reading or arithmetic. These relations were as predicted. As these relations have not been examined for children with ADHD, these findings are new. It is to be noted, however, that our findings and interpretations differ from those reported by Glutting et al. (2006) for a normative sample. They reported that the FSIQ accounted for approximately 60% of the variance for both reading and arithmetic scores, and the subscale index scores added less than 1% variance in the predictions.

Our findings have implications for the use of WISC-IV with children with ADHD. As the ECV of a general factor can be interpreted as the degree of unidimensionality of general factor to the specific factors (Reise et al., 2013a), these findings indicate support for utilization of FSIQ scores, but not the subscale scores. As ω_h_ is a measure of internal consistency reliability (Brunner et al., 2012), our findings indicate high level of measurement precision for the FSIQ index, and low precision for the subscale scale scores, thereby adding further support for the utilization of the FSIQ score and not subscale scores (Schwean and McCrimmon, 2008; Flanagan et al., 2013). In this respect, although our findings showed that the WM specific factor predicted reading and arithmetic abilities, its low ECV and the ω_s_ values (0.05 and 0.17, respectively) means that predictions from this factor may not be interpretable. Overall, it can be argued that profile analysis that aims to ascertain strengths and weaknesses on the basis of discrepancies in subtest scores (Wechsler, 2003; Flanagan and Kaufman, 2004) may be of little value. Our recommendation for the use of the FSIQ over the subscale scores is consistent with existing recommendations for children in general (Bodin et al., 2009; Watkins, 2010; Devena et al., 2013; Golay et al., 2013; Nakano and Watkins, 2013; Watkins et al., 2013; Canivez, 2014; Styck and Watkins, 2016). Since this recommendation is based indirectly via support for the bifactor model, such practice needs to ensure that there will be no bias in the FSIQ score. According to Reise et al. (2013b) this can be assumed if the ECV and ω_h_ values of the general factor are ≥0.60 and ≥0.70, respectively. As this was the case for the general factors in both the WISC/bifactor and CHC/higher order factor models, it follows that the FSIQ score will not be biased.

Although we have argued in favor of the FSIQ score, the study findings showed a relatively high ω_s_ value for the PS subscale, with both its subtests (Coding and Symbol Search) having higher factor pattern coefficients on the specific factor than the general factor. These findings raise the possibility that the PS could, in part, provide a measure of abilities in PS that is not captured by the FSIQ.

There are limitations in this study that need to be considered when interpreting the findings and conclusions in this study. First, all the participants in this study were from the same clinic, and did not constitute a random sample. Thus, it is possible that this may constitute a bias for the sample examined, limiting the findings and conclusions made in this study to ADHD in general. At a practical level, however, it is difficult and virtually impossible to obtain random samples involving clinical samples. Indeed, the previous studies that have examined the factor structure of the WISC-IV in children with ADHD have not used random samples (Yang et al., 2013; Styck and Watkins, 2014; Thaler et al., 2015). Second, as it is possible that as our sample, like previous studies in this area, was highly heterogeneous in terms of psychopathology, the findings may have been confounded. Although there is evidence that sample heterogeneity could potentially influence the results of the factor analysis in general (Delis et al., 2003), Devena et al. (2013) found no difference in factor analysis of the WISC-IV between their full sample and a subsample of this sample that excluded children without disabilities and ADHD. Thus it is possible that sample heterogeneity may not be a confounding variable in relation to WISC-IV scores. Third, in the current study the factor pattern coefficients of the subtests in the WISC/bifactor model with the WM and PS factors were constrained equal, as this model was otherwise empirically under-identified. Thus, the findings may have been confounded. Although we have highlighted a number of limitations, we believe that the findings in the current study add to the literature on the structural model of the WISC-IV and for children with ADHD and children in general, and also the adequacy of using the FSIQ score (over the scale scores) for research and clinical practice. It would be useful if more studies were conducted in this area, taking into consideration the limitations highlighted here.

## Author Contributions

RG: Conception and design of research; analysis and interpretation of data; drafting and revising manuscript; approval of final manuscript; accountability for accuracy and integrity of work. AV: conception and design of research; acquisition, analysis, and interpretation of data; drafting and revising manuscript; approval of final manuscript; accountability for accuracy and integrity of work. SW: conception and design of research; analysis and interpretation of data; drafting and revising manuscript; approval of final manuscript; accountability for accuracy and integrity of work.

## Conflict of Interest Statement

The authors declare that the research was conducted in the absence of any commercial or financial relationships that could be construed as a potential conflict of interest.
